# Supplementary material for: Green synthesis of propylene oxide directly from propane
Source: Nat Commun. 2022 Dec 13;13:7504. doi: 10.1038/s41467-022-34967-2 (PMC9748031; doi:10.1038/s41467-022-34967-2)
Supplement: Supplementary file 1 — Supplementary Information [file 41467_2022_34967_MOESM1_ESM.pdf]

Supplementary Information for

Green synthesis of propylene oxide directly from propane

Pierre Kube,[1] Jinhu Dong,[1] Nuria Sánchez Bastardo,[2] Holger Ruland,[2] Robert Schlögl,[1,2]

Johannes T. Margraf,[3] Karsten Reuter,[3] and Annette Trunschke[1,*]

[1]*Fritz-Haber-Institut der Max-Planck-Gesellschaft, Department of Inorganic Chemistry, Faradayweg 4-6, 14195 Berlin (Germany).*

[2]*Max-Planck-Institut für Chemische Energiekonversion, Department of Heterogeneous Reactions, Stiftstrasse 34-36, 45470 Mülheim an der Ruhr (Germany).*

[3]*Fritz-Haber-Institut der Max-Planck-Gesellschaft, Theory Department, Faradayweg 4-6, 14195 Berlin (Germany).*

kube@fhi-berlin.mpg.de
jhdong@fhi-berlin.mpg.de
nuria.sanchez-bastardo@cec.mpg.de
holger.ruland@cec.mpg.de
rs01@fhi-berlin.mpg.de
margraf@fhi.mpg.de
reuter@fhi.mpg.de
corresponding author: trunschke@fhi-berlin.mpg.de

Supplementary Table 1: Properties of the materials filled into the reactor.

	S_{BET} (m^2 g^{-1})	V_p (cm^3 g^{-1})	Impurities (wt-%)	Phase composition	Themal conductivity (W $m^{-1}K^{-1}$)
SiO_2	1.6	0.0004	0.014 Ti<br>0.003 Mn	α-quartz	7.7-8.4
Aerosil 380	394	1.8	-	Amorphous	0.02
SiC	0.1	0.0006	0.007 Ti<br>0.002 Mn	mixture of various SiC polytypes<br>main component: moissanite-6H	32-270
h-BN	9.0	0.005	0.7 Si<br>0.4 Ca<br>0.2 Zr<br>(0.04) Cr	Hexagonal	220-420

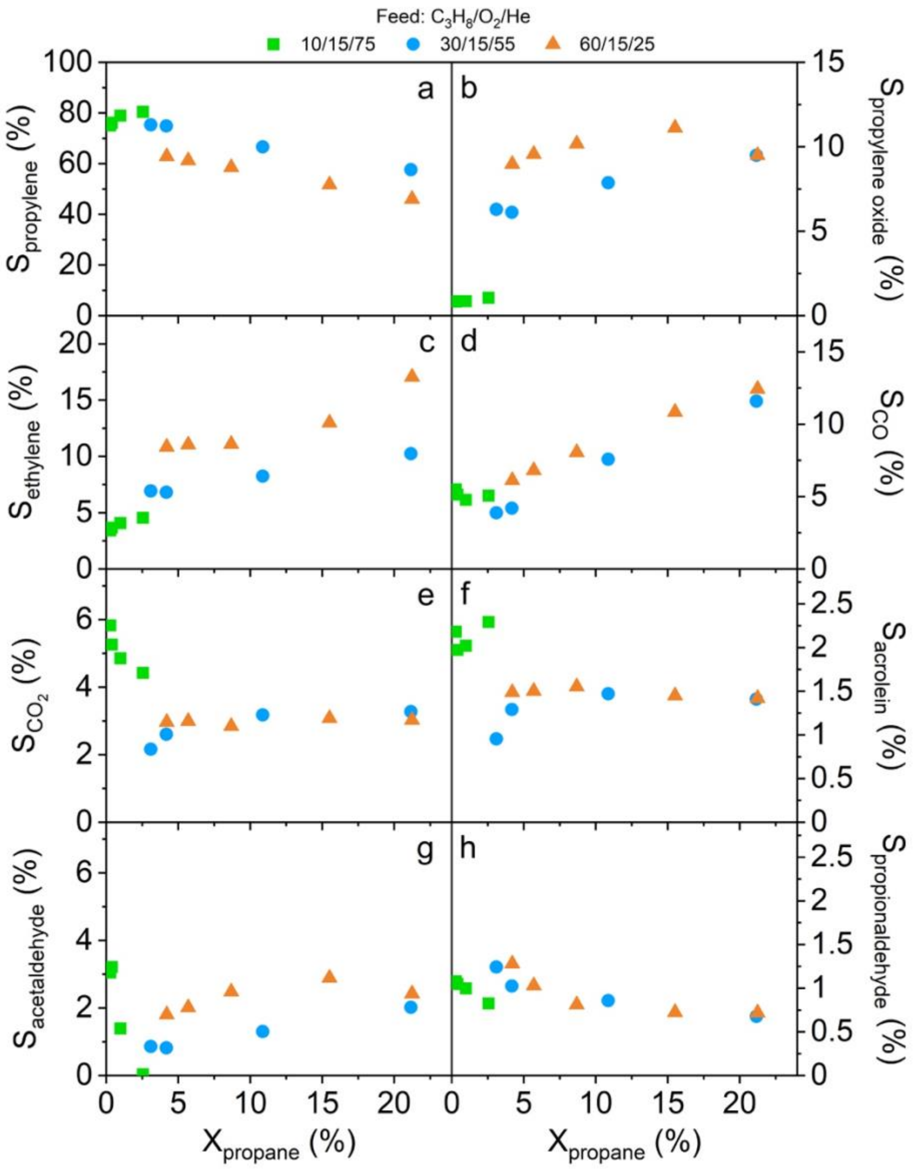

Supplementary Fig. 1: Product selectivity in the oxidation of propane using three different feeds over SiO_2.

Selectivity to **a**, propylene, **b**, propylene oxide, **c**, ethylene, **d**, CO, **e**, CO_2, **f**, acrolein, **g**, acetaldehyde, and **h**, propionaldehyde measured for three different feed compositions as indicated in the legend on top; Reaction conditions: Mass of filling material = 666 mg, T= 490°C, W/F = 1.6 to 6.0 g s ml^{-1}, feed (C_3H_8/O_2/He) = 10/15/75 (square symbols), 30/15/55 (circle symbols), 60/15/25 (triangle symbols); Conversion of O_2 reaches 100% at W/F = 6.0 g s ml^{-1}.

Supplementary Table 2: Apparent activation energies measured in the present work and for the same and different feed compositions in the literature.

	$E_{a,propane}$ (kJ mol^{-1})	Feed composition (C_3H_8/O_2/Inert)
h-BN	230 +/-8	30/15/55
SiO_2	286 +/-21	30/15/55
Aerosil 380	269 +/-8	30/15/55
SiC	323 +/-38	30/15/55
Quartz wool	297 +/-14	30/15/55
BNOH[1]	184	1/1.5/3.5
h-BN[2]	192	1/1/9
h-BN[3]	180	1/1.5/3.5
h-BN[4]	253	30/15/55

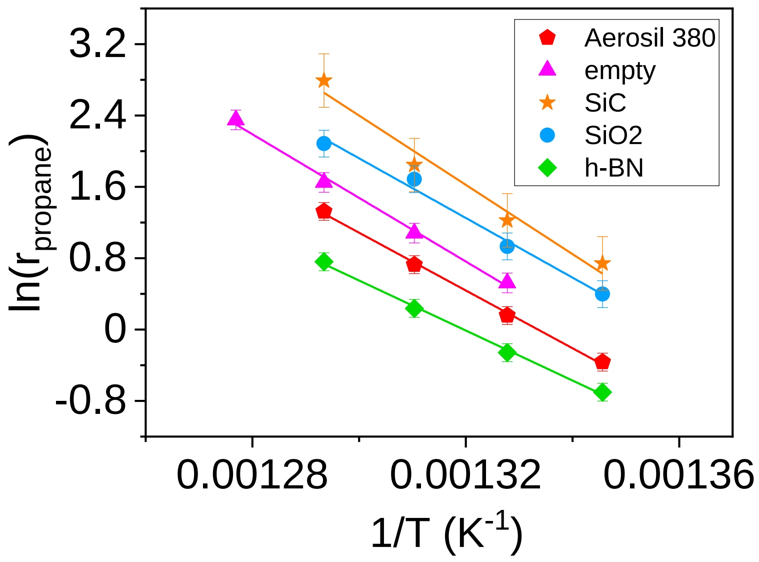

Supplementary Fig. 2: Arrhenius plot for the determination of the apparent activation energy for the used materials.

Reaction conditions: T = 470 °C - 510 °C, total flow = 10 ml min^{-1}, feed (C_3H_8/O_2/He) = 30/15/55; The error bars result from 5 chromatographic measurements.

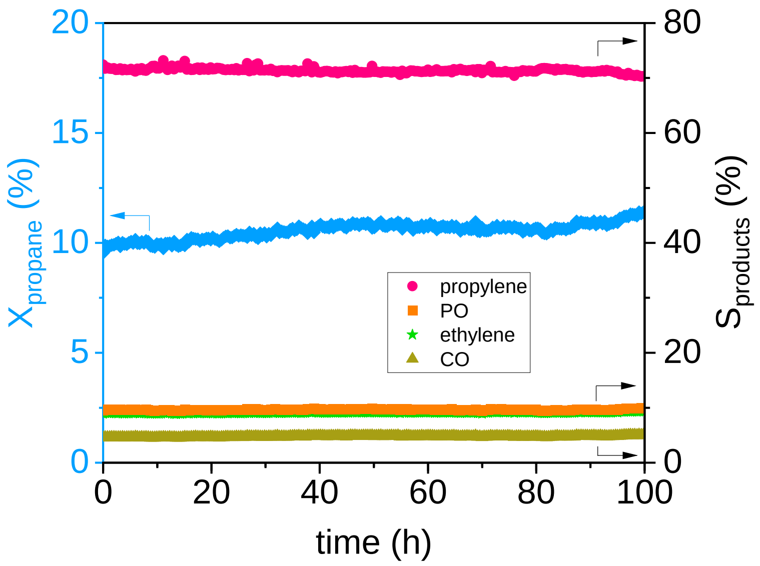

Supplementary Fig. 3: Conversion of propane and selectivity to the products (see legend) over silica (α-quartz).

Reaction conditions: T = 500°C, feed ($C_3H_8/O_2/He$ = 30/15/55), total flow rate (10 ml/min), m = 0.666 g.

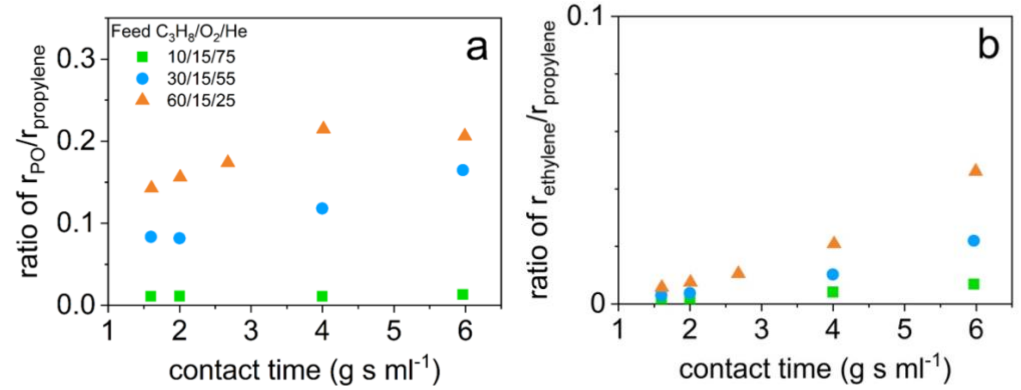

Supplementary Fig. 4: Impact of the propane concentration on the reaction rates.

a, Ratio of $r_{PO}/r_{propylene}$ and, **b**, $r_{ethylene}/r_{propylene}$ measured for SiO_2 as a function of W/F at 490°C in three different feeds as indicated in the legend; Reaction conditions: Mass of SiO_2 = 666 mg, feed ($C_3H_8/O_2/He$) = 10/15/75, 30/15/55, and 60/15/25.

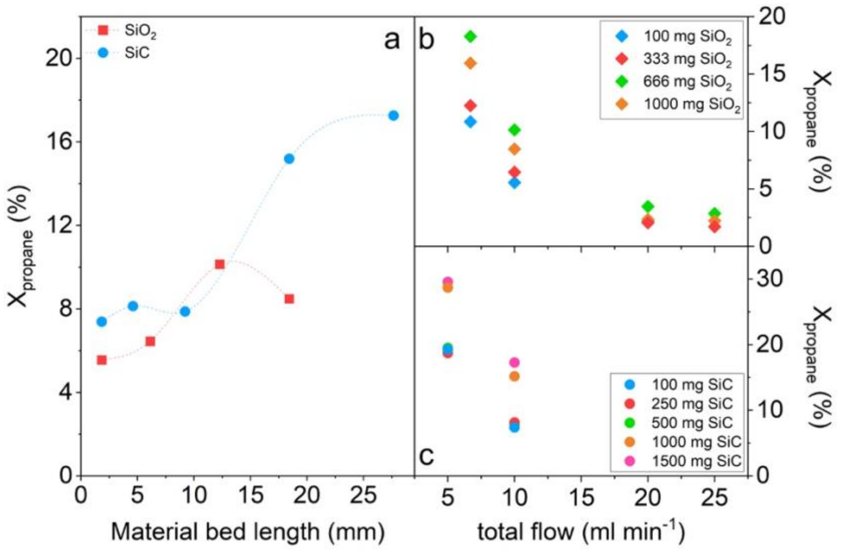

Supplementary Fig. 5: Analysis of the contribution of interfacial reactions and diffusion limitations.

a, Propane conversion as a function of the material bed hight; Reaction conditions: T = 490°C, F = 10 ml min^{-1}, feed ($C_3H_8/O_2/He$) = 30/15/55; Conversion of propane as a function of the total flow measured with **b**, SiO_2 and, **c**, SiC as filling materials; Reaction conditions: T = 490°C, flow = 4 to 25 ml min^{-1}, feed ($C_3H_8/O_2/He$) = 30/15/55.

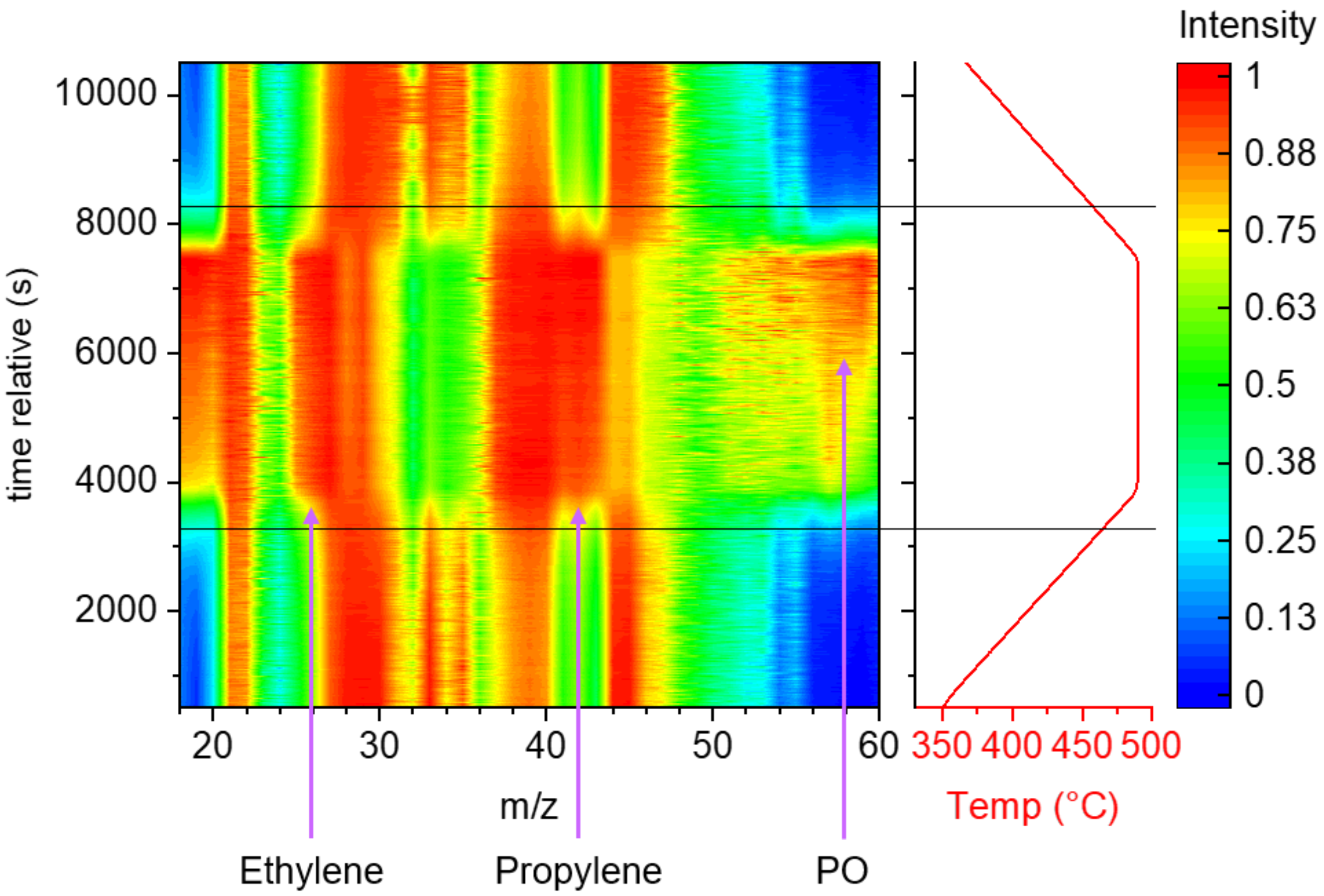

Supplementary Fig. 6: Temperature programmed reaction using *h*-BN as filler.

Reaction conditions: T = 350 °C - 490 °C, total flow = 10 ml min^{-1}, m = 665 mg, feed ($C_3H_8/O_2/He$) = 30/15/55, heating rate 2.5 K min^{-1}; Mass-to-charge ratios m/z 18 to 60 were recorded.

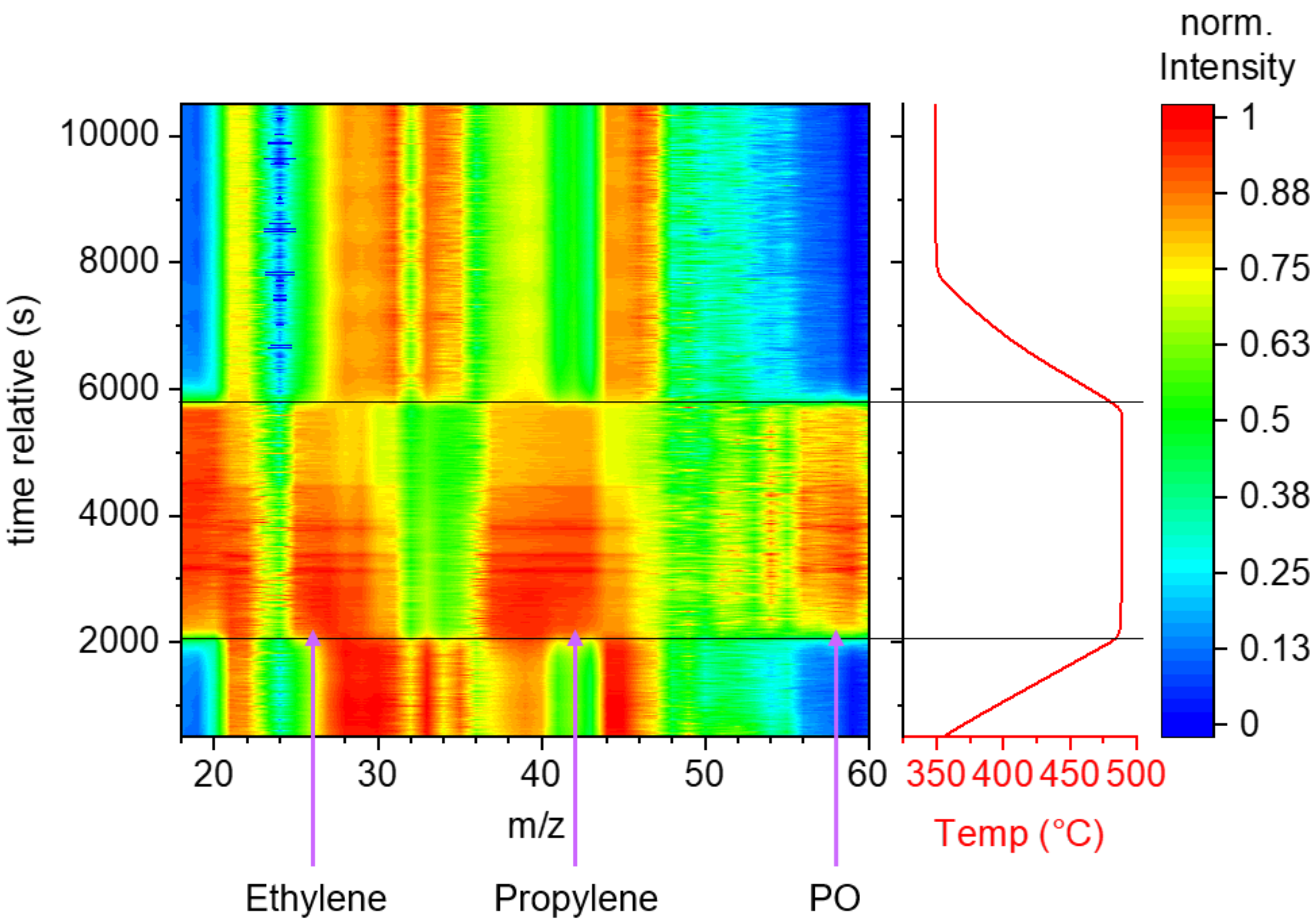

Supplementary Fig. 7: Temperature programmed reaction using SiO_2 as filler and faster heating rate.

Reaction conditions: T = 350 °C - 490 °C, flow = 10 ml min^{-1}, m = 670 mg, feed ($C_3H_8/O_2/He$) = 30/15/55, heating rate 5 K min^{-1}; Mass-to-charge ratios m/z 18 to 60 were recorded.

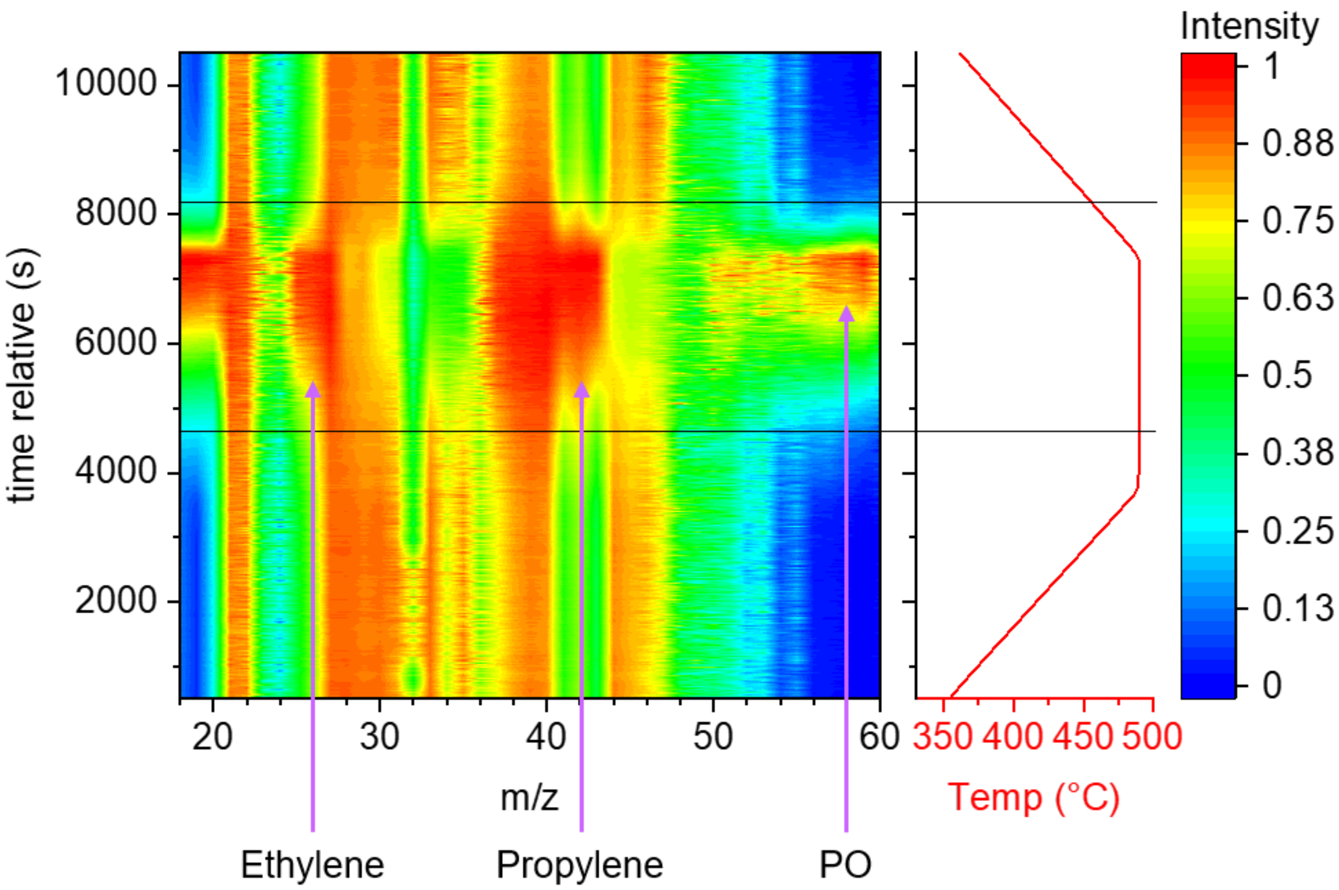

Supplementary Fig. 8: Temperature programmed reaction in an empty reactor.

Reaction conditions: T = 350 °C - 490 °C, flow = 10 ml min^{-1}, feed ($C_3H_8/O_2/He$) = 30/15/55, heating rate 2.5 K min^{-1}; Mass-to-charge ratios m/z 18 to 60 were recorded.

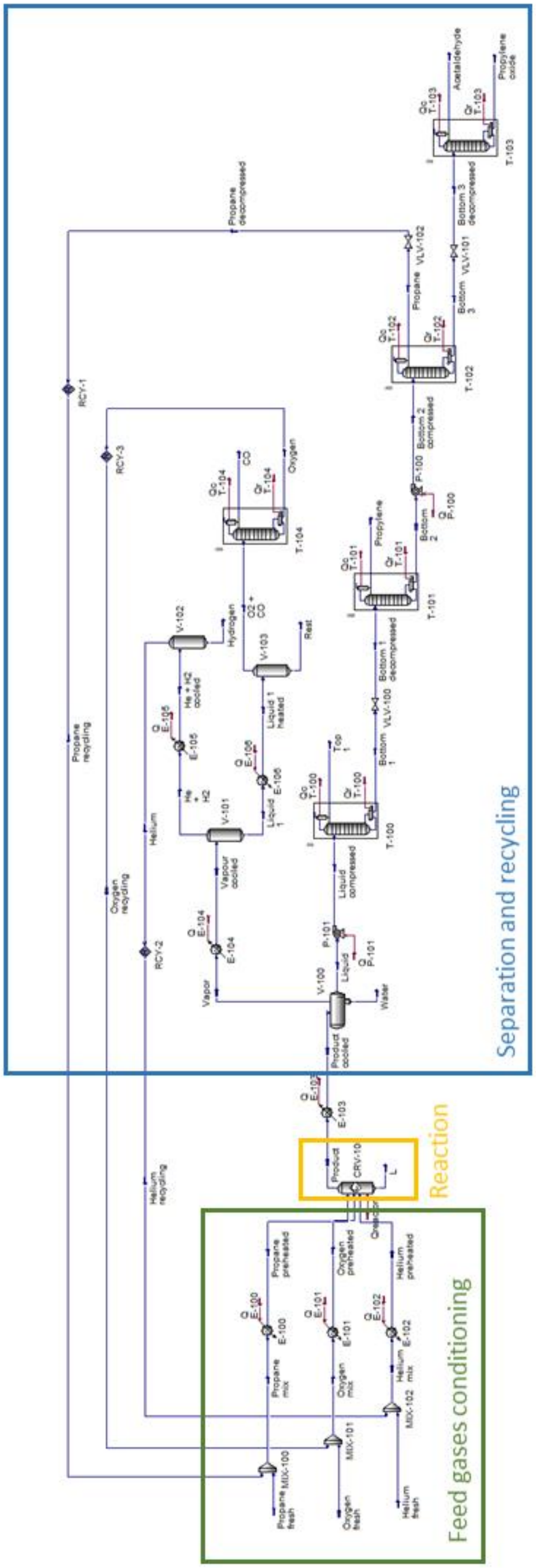

Supplementary Fig. 9: Aspen HYSYS flow-sheet for direct oxidation of propane to propylene oxide.

Supplementary Table 3: Global recovery and mole fraction of the final streams.

Stream in PFD	Global recovery (%)[a]	Mole fraction
Propane	95.73	0.9990
Propylene oxide	99.76	0.9900
Acetaldehyde	96.53	0.9548
Hydrogen	98.63	0.9978
Oxygen	94.59	0.9900
Propylene	94.93	0.9900
Helium	99.97	0.9984

[a] Global recovery of comp. *i* (%) = Mole flow of comp. *i* in the stream *i* / Mole flow of comp. *i* in *Product* x 100

Supplementary Table. 4: Economic analysis of the combined process for propylene and propylene oxide production.

How many years to profit	5	years
Total Capital Cost	17,221,700	$
Total Operating Cost	13,974,700	$/year
Total costs in 5 years	87,095,200	$
Total profit from Hydrogen + Acetaldehyde + Propylene in 5 years	44,234,011	$
Difference (Investment - Profit)	42,861,189	$
Total propylene oxide production in 5 years	12,005	ton
Price of propylene oxide from propane dehydrogenation + propene epoxidation	3,570	$/ton
Commercial price of PO	2,807	$/ton

References

1 Shi, L. *et al.* Edge-hydroxylated Boron Nitride for Oxidative Dehydrogenation of Propane to Propylene. *ChemCatChem* **9**, 1788-1793, doi:10.1002/cctc.201700004 (2017).

2 Tian, J. S. *et al.* Hexagonal boron nitride catalyst in a fixed-bed reactor for exothermic propane oxidation dehydrogenation. *Chem. Eng. Sci.* **186**, 142-151, doi:10.1016/j.ces.2018.04.029 (2018).

3 Chen, J. J. *et al.* Boron-hyperdoped silicon for the selective oxidative dehydrogenation of propane to propylene. *Chem. Commun.* **56**, 9882-9885, doi:10.1039/d0cc02822c (2020).

4 Grant, J. T. *et al.* Selective oxidative dehydrogenation of propane to propene using boron nitride catalysts. *Science* **354**, 1570-1573, doi:10.1126/science.aaf7885 (2016).
