# Supplementary material for: Green synthesis of propylene oxide directly from propane
Source: Nat Commun. 2022 Dec 13;13:7504. doi: 10.1038/s41467-022-34967-2 (PMC9748031; doi:10.1038/s41467-022-34967-2)
Supplement: Supplementary file 3 — Description of Additional Supplementary Files [file 41467_2022_34967_MOESM3_ESM.pdf]

Description of Additional Supplementary Files

File Name: Supplementary Software 1
Description: The Python code for running the microkinetic simulations with the open-source Cantera package is provided at https://ac.archive.fhi.mpg.de/P51805.
